# Case Report: Metagenomics Next-Generation Sequencing Can Be Performed for the Diagnosis of Disseminated Mucormycosis

**DOI:** 10.3389/fmed.2021.675030

**Published:** 2021-10-22

**Authors:** Yi Sun, HuiLing Li, JiaJun Chen, ZhongHui Ma, Pin Han, YuChen Liu, Jing Wen, Fang Ren, XiaoXu Ma

**Affiliations:** ^1^Department of Gynecology, The First Affiliated Hospital of Zhengzhou University, Zhengzhou, China; ^2^Gene Hospital of Henan Province, Precision Medicine Center, The First Affiliated Hospital of Zhengzhou University, Zhengzhou, China; ^3^Department of Respiratory, The First Affiliated Hospital of Zhengzhou University, Zhengzhou, China; ^4^School of Public Health, Zhengzhou University, Zhengzhou, China; ^5^Department of Stomatology, The First Affiliated Hospital of Zhengzhou University, Zhengzhou, China

**Keywords:** mucormycosis, diabetes, pulmonary cavity, alveolar lavage, metagenomics next generation

## Abstract

Mucormycosis is an infection caused by a group of filamentous molds with in the order Mucorales. In developing countries, most cases of mucormycosis occur in persons with poorly controlled diabetes mellitus or subjects with normal post-traumatic immune function. Mucormycosis exhibits a marked propensity for invading blood vessels. The mortality rate of invasive mucormycosis is very high (>30–50%), and 90% of mortality is related to disseminated diseases. We report a 62-year-old man with underlying diseases, such as diabetes and psoriatic arthritis, with a history of trauma before admission. Chest CT showed multiple cavities. Based on the suspected clinical manifestation of mucormycosis infection, the patient received a microbiological culture of bronchoalveolar lavage fluid, and metagenomics next generation sequencing (mNGS) was performed. The results suggested *Klebsiella pneumoniae* infection. However, *Rhizopus microsporus* strains were shown by the mNGS of transpulmonary puncture tissue. Therefore, we report a case in which rare pathogens are identified by mNGS.

## Introduction

*Mucor* is a rare and serious-conditioned pathogenic fungus, and can be found in the oral cavity and nasopharynx of normal people. However, in the presence of high-risk factors, such as diabetes, malignant tumors, agranulocytosis, AIDS, malnutrition, and the use of glucocorticoids (1), mucormycosis has become the third cause of invasive fungal infection after *Aspergillus* and *Candida* (2). The disease progresses rapidly, and the mortality is high after mucormycosis infection. However, its clinical manifestation is not specific, and results of laboratory tests are not specific either. Metagenomics next-generation sequencing (mNGS) refers to the direct extraction of nucleic acids of all microorganisms from clinical or environmental samples, construction of a metagenomic sequencing library, and sequencing. Through mNGS, some microbial species that cannot be cultured or difficult to be cultured are found in samples, and unknown and rare microbial species in complex samples are discovered (3). It has the advantages of high speed and high accuracy.

Herein, we report a case of *Rhizopus* infection with a variety of underlying diseases, such as diabetes and psoriatic arthritis. The clinical manifestations of the patient were not specific, and the underlying diseases were obvious. CT showed that the lung cavity was not specific. At first, *Klebsiella pneumoniae* was diagnosed and treated according to the results of bronchoalveolar lavage, but the effect was not satisfactory. Later, mNGS was performed to detect the lung tissue of the patient, and the presence of *R. microsporus* was quickly found, so the cause was finally determined.

## Case Description

We report a 62-year-old man with mucormycosis who suffered from chronic hepatitis B for 20 years. A month before admission, trauma caused chest pain, wounds on the right upper limb and right knee joint. Six days before admission, there was no obvious inducement for aggravation of joint pain and chest pain. Chest CT was performed locally and showed the shadow of the lungs. The local diagnosis was rheumatoid arthritis, type 2 diabetes, and hepatitis B. He received related treatment in a local hospital. However, his joint pain was not significantly relieved, while coughing and coughing dark red sputum occurred at the same time, so he went to the rheumatic immunology department of our hospital.

**Figure 1:**
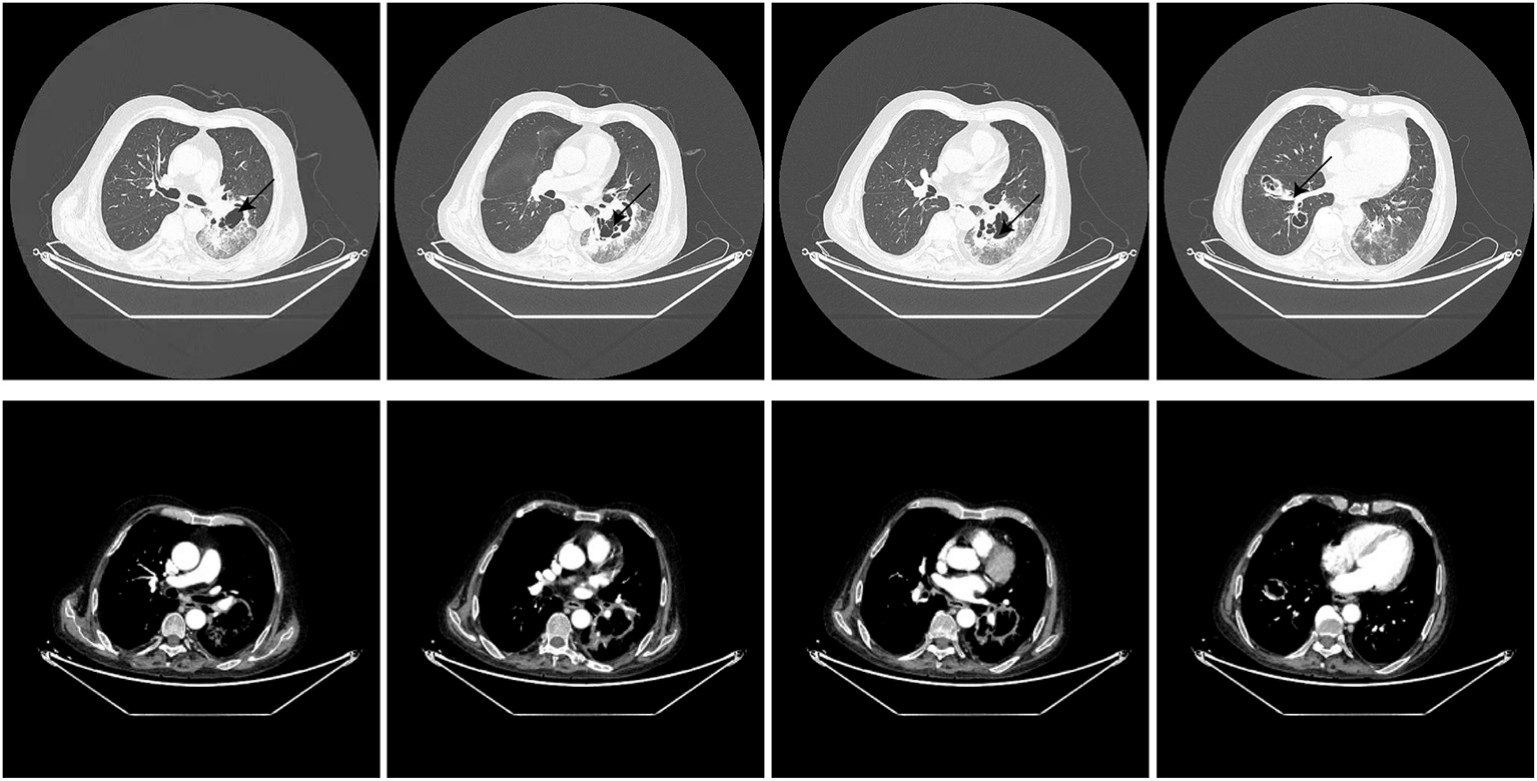
Chest CT upon admission to the hospital.

The vital signs were normal on admission. Physical examination showed that the posterior pharyngeal wall was red and swollen. The shoulder and knee joints were swollen. An irregular wound could be seen on the inside of the upper limb and knee joint. Relevant examinations and tests were improved after admission. Test results are shown in Table 1. Chest CT showed multiple cavities in the lower lobe of both lungs (Figure 1). Therefore, the patient was initially diagnosed with rheumatoid arthritis, pulmonary infection, and hepatitis B. He was given levofloxacin 0.5 g IV once daily (QD) for anti-infective treatment and metformin 50 mg orally (PO) two times daily to control blood sugar.

**Table 1:** Test results after admission.

**Inspection item**	**Actual value**	**Unit**	**Normal value**
WBC	9.60	10^9^/L	3.5–9.5
Neut%	84.0	%	40–75
Lymph%	6.50	%	20–50
PCT	0.40	ng/mL	Suggested antibiotics
CRP	104.83	mg/mL	104.83
(1–3)-β-D-glucan	<10.00	pg/mL	0–100
HumanGlactomannan	0.250	μg/mL	0–0.5
AspergillusGlactomannan	≤0.25	μg/mL	<0.85
Anti-CCP	1162.60	RU/mL	0–25
Anti-MCV	523.82	U/mL	0–30
RFIgG	1:100 (±)		(–)
RF IgM	196.4	IU/mL	0–30
AKA	(+)		(–)
APF	(+)		(–)

Three days after admission, the patient still had a severe cough with dark red sputum. Therefore, he was transferred to the respiratory department for treatment on the third day of admission. The physical examination showed that there was a small amount of wet rale in the left lower lung and a large ulcer in the right upper limb. On the first day after the transfer, a large amount of purulent secretions from the left main bronchus and a small amount of purulent secretions from the right main bronchus were observed by tracheoscopy. Bronchoalveolar lavage fluid (BALF) was collected and tested for microbial culture and mNGS. On the third day after transfer, the patient developed fever, and the highest temperature was 38.5°C, which returned to normal after physical cooling. The fever of the patient was thought to be due to infection. Microbial culture and mNGS of BALF showed *Klebsiella pneumoniae* infection (Table 2). On the fourth day after transfer, according to the results of drug sensitivity, the antibiotic was adjusted to piperacillin sodium 4.5 g IV every eight hours (Q8h). Low blood glucose was detected at this point, so he stopped using metformin. On the seventh day after transfer, a blood routine examination showed that white blood cells were 4.8 × 10^9^/L and C-reactive protein (CRP) was 85.6 mg/L.

**Table 2:** Results of three metagenomics next-generation sequencing (mNGS).

**Sampling tissue**	**Name**	**Sequence number**	**Name**	**Sequence number**	**Attention**
BALF	Klebsiella	54,738	Klebsiella pneumoniae	41,019	High
Skin	Rhizopus	220	Rhizopus microsporus	197	High
Lung tissue	Rhizopus	56	Rhizopus microsporus	48	High

The 9th day after transfer to respiratory department recommended debridement treatment and sent it to mNGS. In the process of waiting for the results, the patient coughed intermittently with grayish-brown sputum. During this period, we continued the anti-infection treatment and maintained good blood glucose control, so we did not continue to use drugs for blood glucose. Finally, the result of mNGS submitted by debridement tissue indicated *R. microsporus* infection (Table 2). Combined with upper limb mNGS and imaging results, the possibility of lung complicated with *Mucor* infection is considered. Therefore, the amphotericin B antifungal therapy was added on the 11th day after transfer.

**Figure 2:**
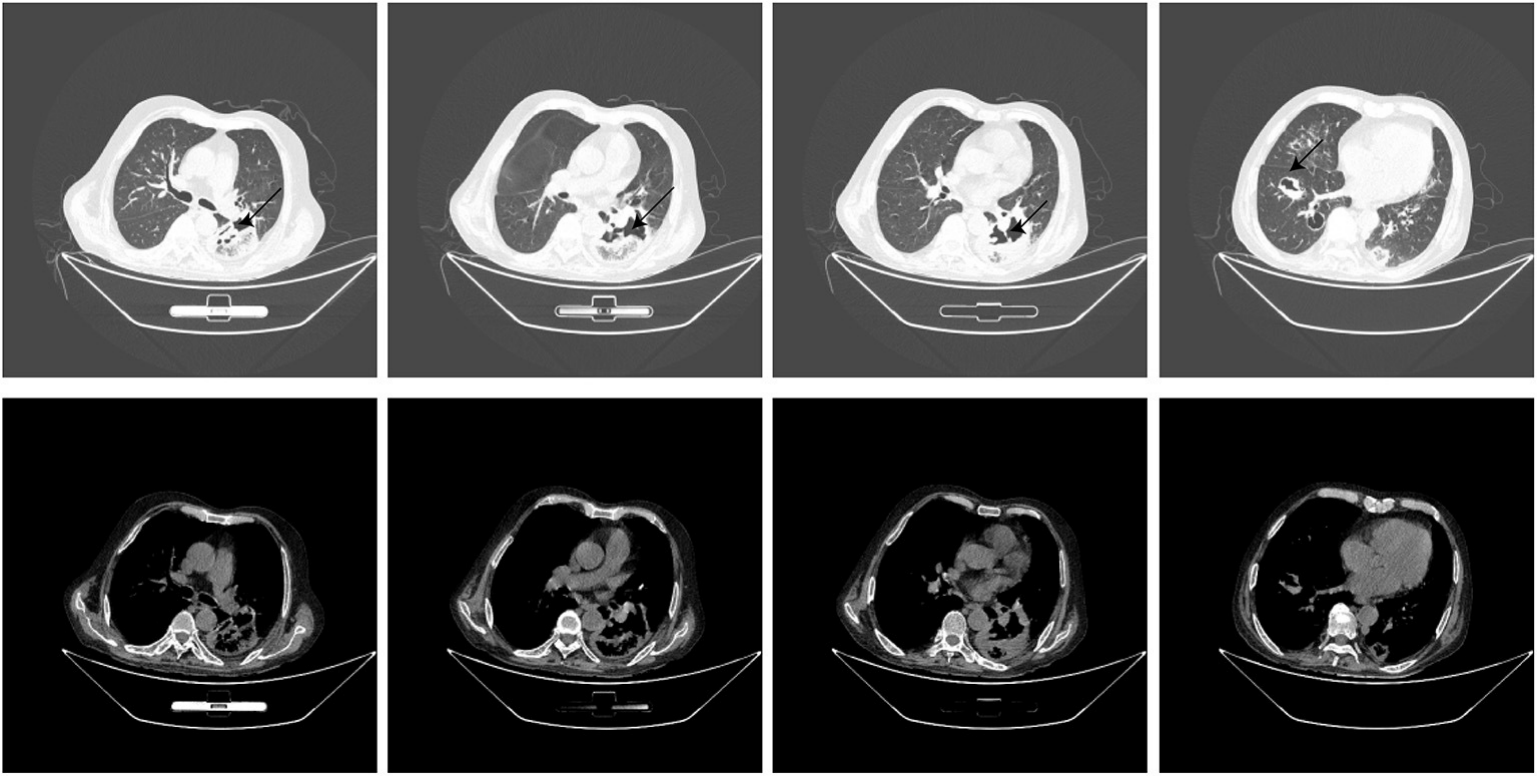
Chest CT 10 days after transfer.

**Figure 3:**
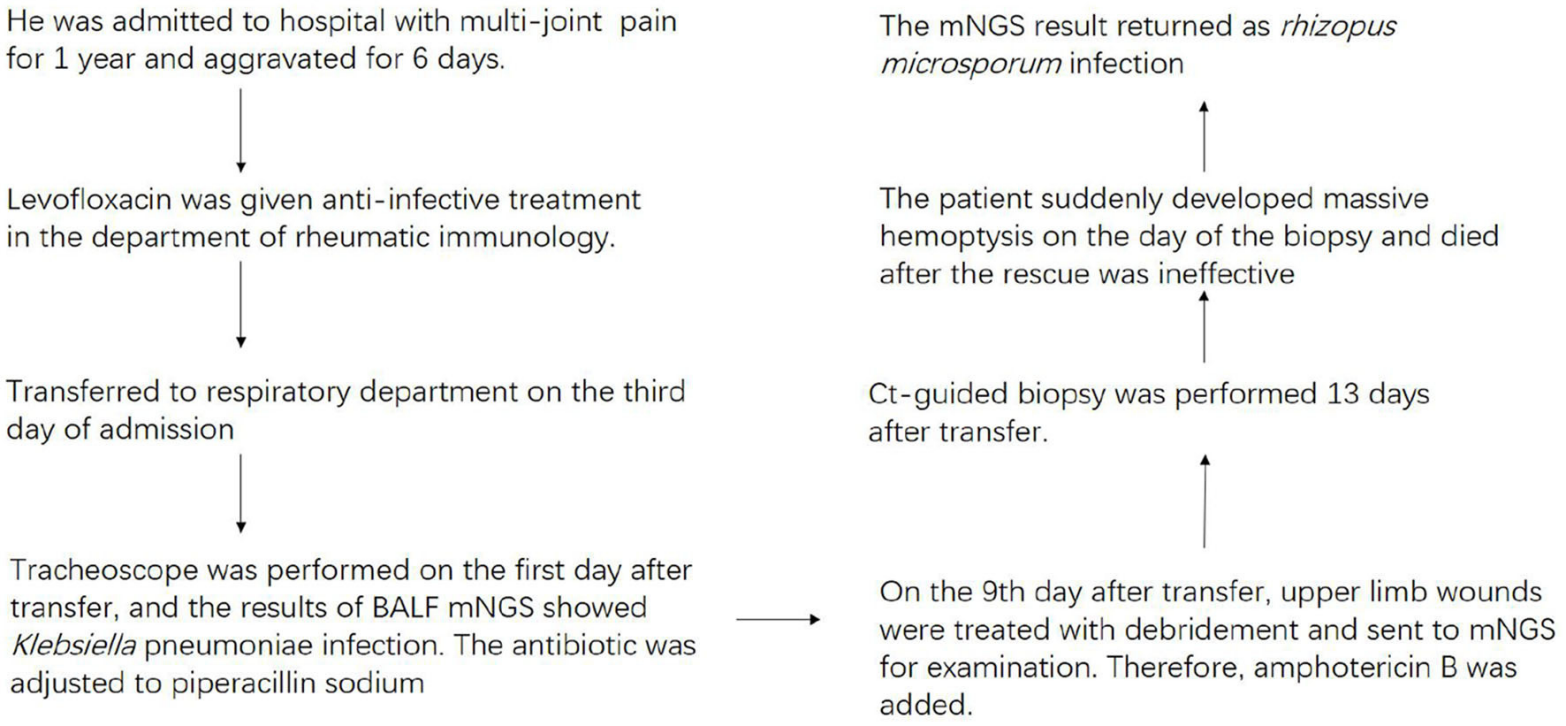
Diagnosis and treatment of the patient.

Thirteen days after being transferred to the respiratory department, the chest CT showed that the lung lesions were worse than before (Figure 2), and the empirical anti-infection treatment was poor. The diagnosis was further confirmed by CT-guided biopsy, and the punctured tissue was sent to pathology for mNGS detection. However, the patient suddenly developed massive hemoptysis on the day of the biopsy and died after the rescue was ineffective. The final pathology showed that a small amount of suppurative exudate with acute fibrinous tissue surrounding lung tissue causing pneumonia. The mNGS result returned as *R microsporus* infection (Table 2), which was consistent with the left upper limb wound test result. A brief summary of the patient's treatment is given (Figure 3).

## Discussion

The incidence of pulmonary mucormycosis reported in different countries is quite different. In developed countries such as those in Europe and America, the incidence rate is about 0.01/100,000–.2/100,000 (4–6), and the incidence rate in developing countries, such as India, is about 14/100,000 (7). Based on anatomic localization, mucormycosis can be classified as one of six forms: rhino-orbital-cerebral mucormycosis (ROCM), pulmonary, cutaneous, gastrointestinal (GI), disseminated, and mucormycosis of uncommon sites. Pulmonary mucormycosis is the second most common of the six types (8).

Patel et al. (9) reported that the mortality rate of mucormycosis, a serious fatal disease, was as high as 61%. Most patients have one or more risk factors, such as diabetes, silver scrap arthritis, and so on. In this case, the patient suffered from hepatitis B for 20 years and took an immunosuppressant regularly. At the same time, the patient also suffered from psoriasis arthritis, which was in a state of gradual aggravation in recent 1 year. More importantly, although the patient was diagnosed with type 2 diabetes after admission, according to the description of the patient, we suspected that he had been suffering from diabetes for many years. Therefore, the patient in this case also had the above several high-risk factors.

In the process of diagnosis and treatment of the patient, we finally diagnosed him with pulmonary *Mucor* infection, *Klebsiella* pneumoniae infection, and skin *Mucor* infection. Although the pathogen was finally identified, the patient eventually died because of an untimely diagnosis. This is mainly because the mNGS test results of the alveolar lavage sent for inspection indicated *Klebsiella* pneumoniae infection. The most common pulmonary infections in diabetes are *Klebsiella* infection and *Mucor* infection (10–12). A typical CT of *Klebsiella pneumoniae* is characterized by large patches with blurred boundaries and locally protruding backward to form a typical “stalactite disease.” Lesions caused by *Klebsiella pneumoniae* can form smooth cavities with smooth walls in a short period of time, and the diameter of cavities is rarely larger than 2 cm (13). Chest CT manifestations of pulmonary mucormycosis are non-specific. The common manifestations are multiple pulmonary nodules (>10), pleural effusion, pulmonary infarction, “anti-halo sign” (14). Eighty-seven percent of images of mucormycosis can appear “anti-halo sign” (15), but this sign is not characteristic.

Mucormycosis is an invasive fungal disease (10, 16). *Mucor* is characterized by its invasiveness of blood vessels, which leads to rapid and disseminated infection (8). It may be because of this characteristic of *Mucor* that the patient died of hemoptysis in a short time. Therefore, a timely diagnosis is extremely important for rapid therapeutic or surgical intervention and for maximizing the survival rate of patients. For *Mucor*, it has no specific serological index (17, 18). At present, the main methods for diagnosing mucormycosis are biopsy and microbial culture (19). However, microbial culture needs to provide appropriate culture conditions. It has the disadvantages of long culture time, complicated operation and low detection rate. More importantly, it even can delay the optimal treatment time. Routine histopathological examination is accurate but time-consuming.

In recent years, molecular biology techniques, mainly Polymerase Chain Reaction (PCR) and mNGS, have been widely used for the diagnosis of *Mucor*. PCR technology is developing rapidly. It has many outstanding advantages, such as specificity, simplicity, and so on (20). At the same time, it cannot be ignored that PCR also has certain limitations. For example, its specificity depends on the design of primers, and its sensitivity is prone to false-positive results after inspection contamination. However, the emergence of mNGS overcomes the shortcomings of the above methods. mNGS can detect almost all DNA or RNA information in a sample at the same time. It does not require pre-guessing or hypothesis about the type of pathogen causing the infection and overcomes the limitations of targeted diagnostic methods such as being time-consuming and labor-intensive, and slow detection process (21–24). However, mNGS also has some limitations. In the early stages of disease treatment, the mNGS of the early alveolar lavage fluid did not detect the *R. microsporus* infection. It may be because of the thick and hard hyphae of *Rhizopus*. It is difficult to lavage it from the lesion through alveolar lavage (25).

*Mucor* normally exists in environmental surroundings, which may cause the false positive diagnostic of mucormycosis. Therefore, we added a negative control in the test. And we have confirmed mucormycosis was exclusively detected in our samples mentioned in this manuscript and not detected in any other clinical samples and negative controls in the same run.

For the treatment of this patient, two points were very important. The control of blood sugar was the basis of the treatment of this patient, and we did it well. The second was the treatment of *Mucor*. For this patient, although we added amphotericin B in time after the skin tissue suggested *Mucor* infection, we did not give an adequate dose. The dosage of amphotericin B is 0.75–1 mg kg^−1^·day^−1^. The initial dose of amphotericin B liposome was 1–5 mg/day, and the dose of amphotericin B liposome was 3–5 mg kg^−1^·day^−1^. At present, posaconazole and esaconazole are also commonly used in clinics (26). The patient eventually died of massive hemoptysis, which has something to do with our untimely treatment. Therefore, for the treatment of *Mucor*, we should be timely and accurate.

## Conclusion

Mucormycosis has the characteristics of rapid progress, high mortality, and difficult diagnosis. Therefore, if *Mucor* infection is considered in a clinic, appropriate samples can be taken as soon as possible and submitted for mNGS for a definite diagnosis of pathogenic microorganisms and accurate treatment. The emergence of this extended-spectrum pathogen screening technology broadens the idea of traditional pathogen detection and provides a promising broad-spectrum screening method for solving difficult problems such as clinical unknown infection.

## Data Availability Statement

Metagenomics whole genome shotgun sequences have been uploaded to EMBL accession: PRJEB41159.

## Ethics Statement

The studies involving human participants were reviewed and approved by the First Affiliated Hospital of Zhengzhou University. The patients/participants provided their written informed consent to participate in this study. Written informed consent was obtained from the individual(s) for the publication of any potentially identifiable images or data included in this article.

## Author Contributions

YS was involved in analyzing data, analyzing diagnosis and treatment, reading literature, and writing manuscripts. XM was involved in diagnosis and treatment, selecting imaging images, and directing the writing of manuscripts. FR directed the writing of the manuscript. HL and JC participate in the diagnosis and treatment of the disease and improve the cases. ZM, PH, YL, and JW help to obtain clinical data and analyze clinical data. All the authors have read and approved the final manuscript.

## Funding

This research was equally funded and supported by Chinese National Science and Technology Major Project 2018ZX10305410, Henan Province Medical Science and Technique Project grant 2018020001, and Henan province postdoctoral research grant 001801005.

## Conflict of Interest

The authors declare that the research was conducted in the absence of any commercial or financial relationships that could be construed as a potential conflict of interest.

## Publisher's Note

All claims expressed in this article are solely those of the authors and do not necessarily represent those of their affiliated organizations, or those of the publisher, the editors and the reviewers. Any product that may be evaluated in this article, or claim that may be made by its manufacturer, is not guaranteed or endorsed by the publisher.
